# Ventral motion parallax enhances fruit fly steering to visual sideslip

**DOI:** 10.1098/rsbl.2020.0046

**Published:** 2020-05-20

**Authors:** Carlos Ruiz, Jamie C. Theobald

**Affiliations:** Department of Biological Sciences, Florida International University, Miami, FL 33199, USA

**Keywords:** parallax, insect vision, optomotor response, optic flow, *Drosophila melanogaster*

## Abstract

Flies and other insects use incoherent motion (parallax) to the front and sides to measure distances and identify obstacles during translation. Although additional depth information could be drawn from below, there is no experimental proof that they use it. The finding that blowflies encode motion disparities in their ventral visual fields suggests this may be an important region for depth information. We used a virtual flight arena to measure fruit fly responses to optic flow. The stimuli appeared below (*n* = 51) or above the fly (*n* = 44), at different speeds, with or without parallax cues. Dorsal parallax does not affect responses, and similar motion disparities in rotation have no effect anywhere in the visual field. But responses to strong ventral sideslip (206° s^−1^) change drastically depending on the presence or absence of parallax. Ventral parallax could help resolve ambiguities in cluttered motion fields, and enhance corrective responses to nearby objects.

## 1. Introduction

Flies execute extremely fast and precise aerial manoeuvres, requiring robust corrective responses to handle deviations from course. They use coherent motion from optic flow fields to countersteer against changes in direction and position during flight [1,2]. For this purpose, background motion is decomposed into translational and rotational components that are processed independently by large-field neurons in the lobula plate in flies [1–6], and arthropods in general [7].

Responding to translational self-motion requires nearby visual features, because image speed on the retina varies inversely with object distance [8]. Rotational image speeds, by contrast, are unaffected by distance. Some flies take advantage of this difference by increasing their sensitivity to translation in the frontolateral and subequatorial regions of their eyes, where perceived objects are usually closer during natural flight, while displacing the perception of rotation to the dorsal region [4]. *Drosophila*, for example, can use celestial cues above for evaluating changes in direction (reviewed by Warren *et al*. [9]), while positional tasks such as groundspeed control or responses to sudden changes in position are mostly based on optic flow below and near the horizon [10–12].

Translational optic flow additionally provides cues about the three-dimensional structure of the surroundings, which manifests as image speed being inversely proportional to object distances [8,13,14]. Flies can process motion patterns from the frontolateral regions of the visual field and use them to gauge distances [15,16], and separate objects from background [17–19], both critical tasks for navigating through cluttered environments. But motion depth cues are present outside of the frontal or lateral visual field. When flying low over patchy vegetation, for example, a wealth of information about the spatial distribution of features is available right underneath. Bees use this for altitude control [20–22], but flies, for some reason, do not [10,23]. Whether this is because they fail to integrate relative motion beneath, or shift attention to frontal areas during forward flight [24], remains unknown.

The recent finding of a neuron (VT1) in the blowfly *Calliphora vicina*, able to encode motion parallax in the forward and sideslip directions below the horizon [25], provides a partial answer to this question. It demonstrates that at least some groups of flies encode parallax in ventral optic flow, and suggests that this trait could be adaptive to flies traversing habitats with obstacles [26] or foraging for resources on the ground. This could be the case in *Drosophila melanogaster*, a slow flier that searches for fallen fruit.

Despite the abundance of derived traits associated with the lobula plate tangential cells (LPT) across groups of flies [27], horizontal system cells (HS) responsible for assessing yaw rotation are relatively conserved between blowflies and fruit flies [28]. It is therefore possible that they also share the ability to perceive and encode incoherent motion below during flight. We set out to test whether parallax affects the optomotor response of fruit flies during visual perturbations in the ventral or dorsal region of the flow field. We used a virtual flight arena to display perturbations, with and without depth cues, and measure optomotor responses of tethered fruit flies.

## 2. Material and methods

### (a) Experimental subjects

**Figure 1.**
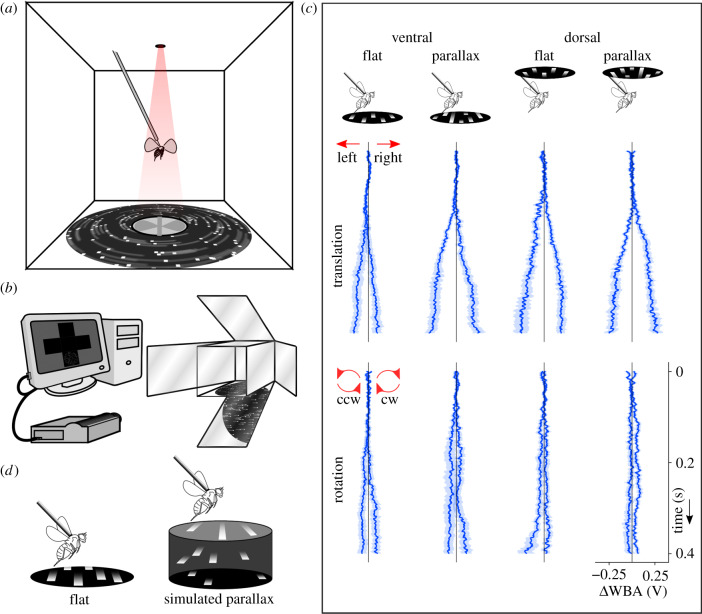
(*a*) Rear view of the projection arena showing the placement of the fly. The IR LED above illuminates the wings in motion casting a shadow on the sensor below the fly. (*b*) The stimulus projects onto the ventral or dorsal faces of a Perspex cube using mirrors. (*c*) Steering attempts are inferred from the difference in the size of shadows of the left and right wings captured by the dual sensor. Mean steering responses of the flies tested can be visualized as time series (solid lines), along with the standard error of the mean (s.e.m.) (shading). (*d*) Motion parallax in the stimuli is simulated by adding relative motion to the dot-field elements.

We tested *D. melanogaster* females within 3–5 days of eclosion, reared in the laboratory under a 12 L : 12 D cycle, kept at 21°C and fed standard medium. Flies were cold anaesthetized, then glued to a fine tungsten rod by the mesonotum. They recovered in the dark for at least 30 min while holding a small piece of paper with their legs, preventing them from flapping their wings. We then removed the paper when suspending each fly in the centre of the arena (figure 1*a*). Each fly was tested only once in an experiment.

### (b) Virtual flight arena

We projected visual stimuli onto the lower and upper surfaces of a 200 mm Perspex cube (figure 1*b*). Perspective-corrected stimuli displayed in a 90° diameter disc. Experiments took place in a dark room to increase contrast, and the sides of the cube prevented flies from getting light from any other direction. Further details of the arena are described in Cabrera & Theobald [29].

### (c) Visual stimuli

Each experiment consisted of open-loop presentations of dot-fields moving either rightward (clockwise in rotation) or leftward (anticlockwise) projected to the ventral or dorsal visual region of the fly (see electronic supplementary material, video S1, for details). Dot-field motion was either rotational or sideslip, at one of four different angular speeds, and with or without parallax depth cues (figure 1*c*). We emulated depth cues by adding relative motion to a randomly distributed group of dots moving in the same direction, suggesting increased distance [12] (figure 1*d*). This ensured the number of dots was constant (113 dots/steradian), and allowed us to add differential speeds to rotational flow fields, which intrinsically have no such feature (see electronic supplementary material, videos S2–S5, for details on the stimuli used). The trials were presented in random order, and interspersed by segments of closed-loop bar fixation to standardize the behavioural state at the beginning of each test [30,31].

### (d) Steering responses

Tethered flies were illuminated from above with an infrared light, while photodiodes below measured the shadow produced by each wing beat. Since flies steer by changing the relative amplitudes of left and right wing beats [32], attempts to turn produce a differential voltage by the sensor pair [33,34], which is reported as the voltage difference in wing beat amplitude (ΔWBA). Responses collected include roll and yaw attempts performed by the fly, as they both result from the same flight mechanics and are indistinguishable using a wing beat analyser [2].

## 3. Results

**Figure 2.**
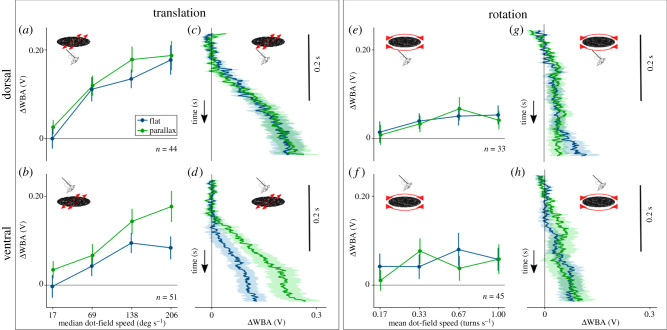
(*a*,*b*) Mean response of *D. melanogaster* to unintended sideslip containing only coherent motion (blue), and with relative motion suggesting the presence of parallax (green). The stimuli were presented at four speeds and two directions on the dorsal and ventral regions of the visual field. Responses were averaged between 0.2 and 0.4 s after stimulus onset. (*c*,*d*) Time series showing the first 0.4 s of the mean response to the highest speed sideslip (206° s^−1^) with and without depth, in the dorsal and ventral visual fields. (*e*,*f*) Mean responses to rotational stimuli with and without relative motion, presented in the dorsal and ventral visual fields. (*g*,*h*) Time series showing the response of the flies to both types of motion at high speed in their dorsal and ventral visual fields. Solid traces represent the mean responses obtained from *n* flies, shading represents s.e.m.

Flies responded to dorsal and ventral stimuli by steering in the direction of the flow, and increasing amplitude with flow speed. Coherent sideways flow with angular speeds up to 138° s^−1^ elicited responses of similar amplitude when presented dorsally and ventrally (figure 2*a*,*b*, blue lines). However, high speeds of sideways flow that suggested stronger disturbances with coherent motion (206° s^−1^) produced significantly weaker ventral responses (*t* = −2.365, *p* = 0.023).

Due to the local optimization of the dorsal region of the eye for evaluating rotation [4], if flies are sensitive to parallax, they might respond to it only when it is present in ventral disturbances. Since motion parallax is exclusive to translation, we expected flies to be unresponsive to it when was added to rotation. For optic flow including relative motion (simulating parallax), response to strong ventral sideslip disturbances increased significantly (*t* = −2.313, *p* = 0.023) (figure 2*d*, green trace). This was similar in amplitude to the response to a dorsal stimulus, either in the absence or presence of depth cues (*t* = −0.001, *p* = 0.999 and *t* = −0.227, *p* = 0.821, respectively) (figure 2*c*). Relative motion had no effect on steering response when added to dorsal sideslip (figure 2*a*, green) or rotation (figure 2*e*,*f*). This occurs even at high-speed translational disturbances presented dorsally (*t* = 0.23, *p* = 0.819) (figure 2*a*, green), or rotational in both regions of the visual field (figure 2*g*,*h*, green) [35].

## 4. Discussion

### (a) Response to dorsal and ventral sideslip disturbances without depth cues

In contrast with hawkmoths *Manduca sexta*, that maintain flight control even with the ventral region of their eyes covered [36], steering responses to positional changes in flies may be strongly based on flow fields below the horizon, as demonstrated in blowflies [4]. In fact, flies respond weakly to translational cues present only in the upper visual hemisphere [12]. However with the narrower dorsal and ventral visual fields shown here, low-speed disturbances containing only coherent motion elicited similar steering responses in both of these regions. Only high-speed disturbances caused a strong difference in the weakening of ventral perturbations without parallax.

Similar steering response shifts are seen during forward flow. Flies shift attention to anterior regions of the ventral flow field as forward flow speed increases [24]. This may alleviate motion blur [37] by focusing attention on areas with slower optic flow, potentially reducing responsiveness to perturbations below. Fast optic flow can also induce spatial summation, forcing the fly to spatially pool information in lateral regions. This increases the ability to respond to fast stimuli at the cost of spatial resolution [38]. It is unknown if sideways perturbations can trigger such neural strategies.

### (b) Response to wide-field incoherent motion

As expected, relative motion cues affected steering responses only in sideslip, and not yaw rotation. Since rotational and translational components of motion are processed separately [1,3,4], encoding relative motion may be a property of large-field neurons, responsible for the translational components of self-motion only. Further, the similarity in the responses to translating dot-fields with and without relative motion in the dorsal region of the eye suggests the presence of a less specialized system for the perception of translation there, in contrast with regions near the horizon where translational cues are more relevant [12]. While incoherent motion is known to be informative in frontolateral regions [15,16], our results extend that range to the ventral region, showing that fruit flies perceive, encode and use depth cues below them. Due to the conservation of traits inherent to the perception of rotation between blowflies and fruit flies [28], we believe elements involved in the integration of depth cues in the ventral flow field in *D. melanogaster* may be homologous to those found by Longden *et al.* [25] in *C. vicina*. Due to strong selective pressures acting on LPT cells [27], the ability to perceive ventral parallax in *D. melanogaster* is a selective trait, with a variety of possibly adaptive roles.

### (c) Height control

Although the depth cues from the ventral optic flow could be used for height control when flying over structured environments (bees are an example [20–22,39,40]), fruit flies seem to control their height during flight using information from frontolateral areas of the visual field [10,23], while referring to ventral optic flow in order to control groundspeed [10,41]. Because the effect of relative motion was notable only during strong sideslip, ventral parallax is probably not involved in altitude control, but further experiments with different levels of parallax would be required to rule it out completely.

### (d) Dealing with ambiguity

Different types of self-motion can generate identical flow fields when perceived by small regions of the eye [4,42], and partial stimulation of wide-field neurons could therefore be ambiguous. In our experiment, for example, moving dots in the small ventral visual field could be perceived by the fly as either sideslip or a roll. However, the presence of parallax in the ventral optic flow could confirm that an otherwise ambiguous stimulus results from translation, because incoherent motion is absent from rotation. However, this is complicated because VS neurons sensitive to roll branch out laterally instead of ventrally on the eye in blowflies [43–46], suggesting that lateral motion on a small region of the ventral field is possibly perceived only as translational, which could also apply to *Drosophila*.

### (e) Navigating complex environments

Our results suggest the presence of relative motion ventrally, even in a narrow cone of vision, is enough to prevent attention from shifting forward, and keep the fly responsive to potential risks below during strong perturbations. The sense of nearness produced by motion parallax induces stronger corrective responses to sideslip disturbances in fruit flies [29]. The fact that fruit flies share the ability to encode parallax information from ventral flow fields with blow flies is not that surprising. The saprophagous nature of both flies forces them to move around in search of ephemeral resources that can be far apart and usually at ground level. While *C. vicina* is a fast flier that moves across patches of differently structured vegetation [26], *D. melanogaster* can forage longer distances and even migrate if necessary in search of resources [47]. With such a natural history, both species can certainly benefit from being aware of the dangers below when traversing unknown structured environments.

We have demonstrated that fruit flies respond to the presence of parallax during strong sideways disturbances in their ventral optic flow. The robustness of this response suggests that it is an adaptive trait, but its full significance is unresolved.

## Supplementary Material

Projection arena

## Supplementary Material

Sideslip stimulus without relative motion (flat)

## Supplementary Material

Sideslip stimulus with relative motion (emulated parallax)

## Supplementary Material

Rotational stimulus without relative motion

## Supplementary Material

Rotational stimulus with relative motion

## Data accessibility

Data are available from the Dryad Digital Repository: https://doi.org/10.5061/dryad.2jm63xskb [35].
